# Cerebral Venous Air Embolism due to a Hidden Skull Fracture Secondary to Head Trauma

**DOI:** 10.1155/2015/730808

**Published:** 2015-11-26

**Authors:** Ai Hosaka, Tetsuto Yamaguchi, Fumiko Yamamoto, Yasuro Shibagaki

**Affiliations:** ^1^Department of Neurology, Hitachinaka Medical Education and Research Center, University of Tsukuba Hospital, 20-1 Ishikawa-cho, Hitachinaka, Ibaraki 312-0057, Japan; ^2^Department of Neurology, Hitachi Ltd. Hitachinaka General Hospital, 20-1 Ishikawa-cho, Hitachinaka, Ibaraki 312-0057, Japan

## Abstract

Cerebral venous air embolism is sometimes caused by head trauma. One of the paths of air entry is considered a skull fracture. We report a case of cerebral venous air embolism following head trauma. The patient was a 55-year-old man who fell and hit his head. A head computed tomography (CT) scan showed the air in the superior sagittal sinus; however, no skull fractures were detected. Follow-up CT revealed a fracture line in the right temporal bone. Cerebral venous air embolism following head trauma might have occult skull fractures even if CT could not show the skull fractures.

## 1. Introduction

Cerebral venous air embolism occurs mostly because of air entry into the brain veins due to some mechanism, including trauma [1–5], central venous catheterization [6, 7], epidural catheterization [8], and administration through the chest drainage tube [9]. In most cases of cerebral venous air embolism caused by head trauma, computed tomography (CT) scan showed the skull fractures and the air might enter through the fractures. We report a case of cerebral venous air embolism in which the skull fracture caused by head trauma was not detected on first CT scan of the head.

## 2. Case

**Figure 1:**
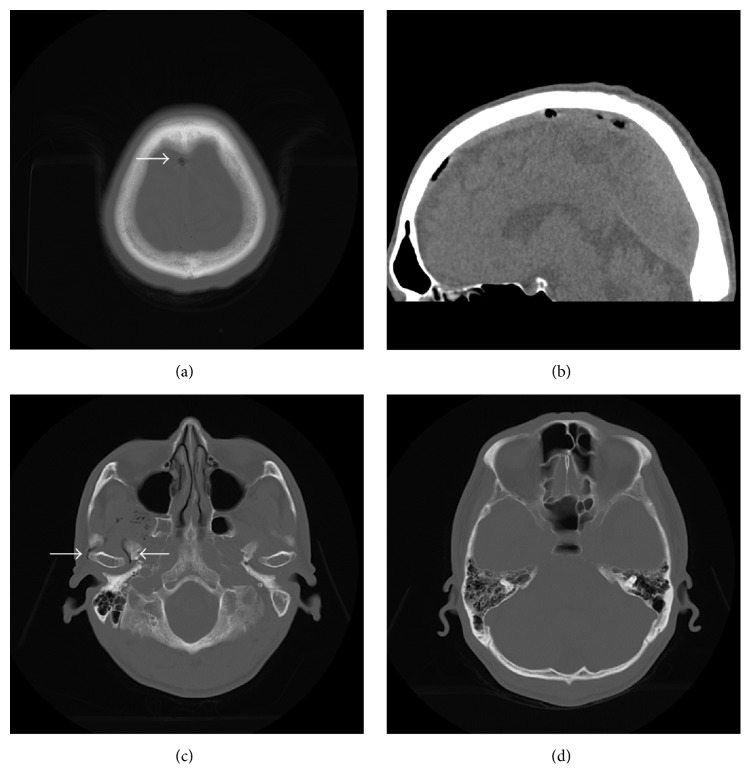
CT scan of the head without contrast: (a) axial cut (bone window) shows air in the superior sagittal sinus (arrow); (b) midsagittal cut of the reconstructed CT (brain window) shows air in the superior sagittal sinus; (c) axial cut (bone window) shows air around the right mandible (arrow); (d) axial cut (bone window) cannot show obvious fractures.

**Figure 2:**
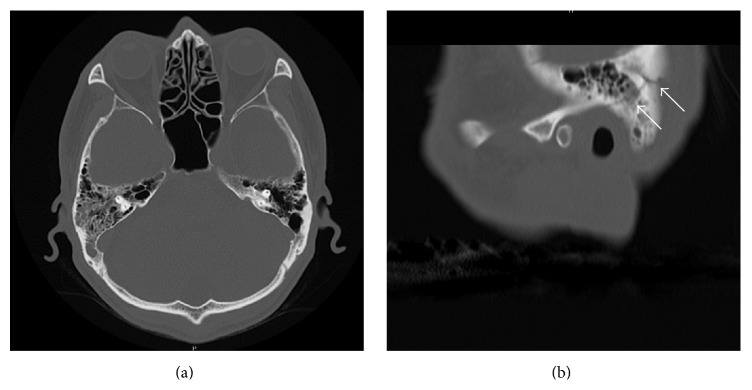
CT scan of the head without contrast 12 days after trauma: (a) axial cut (bone window) cannot show obvious fractures; (b) sagittal cut of the reconstructed CT demonstrates a fracture line in the right temporal bone (arrow).

A 55-year-old man hit the back of his head when he fell and then vomited. He was urgently transported to the hospital. He was alert and had a feeling of fullness in the right ear on arrival; however, there was neither definite hearing loss nor neurologic abnormalities nor cerebrospinal fluid fistula. Helical and nonhelical CT of the head (each 5 mm in slice thickness) were performed. The CT indicated the presence of air in the superior sagittal sinus (Figures 1(a) and 1(b)) and around the right mandible (Figure 1(c)). Bleeding and skull fractures were not detected on the CT images (Figure 1(d)). During the follow-up observation, the neurologic symptoms and fullness in the ear were not exacerbated. Nonhelical CT of the head (each 5 mm in slice thickness) was performed 4 days later. The CT showed that the air in the head disappeared. Helical (each 2.5 mm in slice thickness) and nonhelical (each 5 mm in slice thickness) CT of the head were performed 12 days later. The CT revealed the presence of a fracture line in the right temporal bone (Figure 2).

## 3. Discussion

This case suggested the possibility that a skull fracture is sometimes not detected in cases of cerebral venous air embolism secondary to head trauma. There are some reports on cerebral venous air embolism secondary to head trauma caused by bullet shot [1], traffic accident [2, 4, 5], and falls [3, 10]. Most of the reports [1–5] indicated the presence of definite skull fractures on first head CT images, and air might enter from those regions. Petridis et al. [10] studied patients who had air entry in the cavernous sinus without any skull fractures after head trauma due to falling and suggested the possibility that air might enter from a peripheral venous cannulation. The patient in our study had no definite skull fractures on the CT images at the initial examination and did not undergo central or peripheral venous catheterization; nevertheless, he developed air embolism in the brain veins. The repeat CT scan revealed a fracture line in the temporal bone, from where air may have entered into this region.

The reasons why the fracture was missed in the first CT are as follows. First, the fracture in our case is so minor that routine CT of the head (each 5 mm in slice thickness) could not detect the fracture. Follow-up CT (each 2.5 mm in slice thickness) could detect it because thinner slice thickness of the CT contributed to the improvement of detectability. Second, reconstructed sagittal CT was helpful to demonstrate the fracture more clearly. If minor fracture that the routine CT cannot detect is suspected, thin slice thickness CT and reconstructed sagittal CT are useful to detect it.

In general, cerebral venous air embolism is a favorable disease. However, a skull fracture may induce serious diseases such as central nervous system infection. Furthermore, cerebral venous air embolism might have “hidden” minor skull fractures like in this case. Therefore, a close follow-up with repeated neurological examination and CT scan should be given to patients with cerebral venous air embolism in whom it is not known whether there is a definite skull fracture, considering the possibility of the presence of occult fractures.

This case suggested the possibility that cerebral venous air embolism secondary to head trauma is sometimes not associated with obvious skull fractures. When cerebral venous air embolism is found after head trauma, a possibility of occult fractures should be considered even if the skull fractures were not detected on CT.
